# 

**Published:** 1888-01-28

**Authors:** 


### SPECIAL NOTICE TO NEWSAGENTS, ADVERTISERS, AND THE TRADE.

### CHANGE OF OFFICE AND PUBLISHER.

Notice is hereby given, that the Office of The Hospital will henceforth be at 38, Old Jewry (Cheapside end), E.C., to which address all communications should be sent. Mr. A. Doig has been appointed Advertising Agent, and Mr. J. H. S. Hanning, Manager and Publisher. All Cheques and Post Office Orders should be made payable to J. H. S. Hanning, 38, Old Jewry, E.C., crossed Lloyds, Barnetts, and Bosanquets Bank (Limited).

### Questions and Answers.

### HOW TO ADMINISTER KOLA IN ALCOHOLISM.

F. O.—The best method of administering kola in alcoholism is in the form of kola-chocolate. A cupful of this at breakfast or supper taken regularly, or a cupful between meals, will produce a beneficial and stimulating effect on the system, replacing the craving for drink, and finally curing the habit. The kolatina for making the beverage can be obtained from Messrs. Thomas Christy and Co., 25, Lime Street, E.C., at a cost of 5*s.* per lb., and is sweetened and pleasantly flavoured.

### GETTING ON IN LIFE.

He only is advancing in life whose heart is getting softer, whose blood warmer, whose brain quicker, whose spirit is entering into living peace. And the men who have this life in them are the true lords or kings of the earth—they and they only.—*Ruskin.*

### The English Illustrated Magazine.

### BOOKS RECEIVED.

*British Discomycetes.* By William Phillips. Published by Kegan Paul, Trench, and Co.—*Collected Essays in Preventive Medicine.* By William Squire, M.D. Published by J. and A. Churchill.—*The Creator and Creation.* By W. H. Dallinger. Published by T. Woolmer.—*On Glycosuria.* By William Squire, M.D. Published by J. and A. Churchill.—*Miscellanea Medica Chirurgica.* By E. L. Hussey. Published by Hart, Oxford.—*Olfactics and the Physical Senses.* By C. H. Piesse. Published by Piesse and Lubin.—*Bumblebee Bogo's Budget.* By a Retired Judge. *Aphorisms.* By John Morley. *The Principles of the Art of Conversation.* By J. P. Mahaffy. *The English Illustrated Magazine.* All published by Macmillan and Co.—*The Hospital Prayer-book.* Containing Prayers for Daily and Occasional Use; also a Short Form of Public Service for Lay Readers in Hospitals. By E. J. Waring, C.I.E., M.D. 2nd Edition. Published by J. and A. Churchill.

### AMUSEMENTS WITH PROFIT FOR ALL AGES.

**Rules.**—All answers must be addressed to the PRIZE EDITOR, THE HOSPITAL, 38, Old Jewry, Cheapside, London, E.C., not later than the *first* post on Thursday next. The full name and address must in all cases be given, but a *nom de plume* may be added for publication. Only one side of the paper must be written on.

### FOR MEN AND WOMEN.

### Work Competition.

It may interest those who engaged in the above competition to know what has been done with the various articles they were good enough to prepare and send in for adjudication.

Altogether there were some twenty-nine competitors, and the articles have been divided between the patients in St. Mary's Hospital and those in the Children's Hospital, Paddington Green.

The prize scrap-book was presented to the Ladies' Charity School for Girls, Powis Square, W.

The adjudication was made as follows:

TO ST. MARY'S HOSPITAL.

Dolls from Mary Blaxland, Condorcet, Roma, Maud Parkinson, Miss Collison.

Miss Cornelius, Illumination; Emma, Panel; Matron Seamen's Hospital, Writing Cases; Daisy, Sketch; Esor, Scrap Book; Miss Lucy Henry, Child's Jacket; M. H., Work Specimen; A. E. Grant, Writing Case; Miss Hain, Knitted Jacket.

TO CHILDREN'S HOSPITAL, PADDINGTON.

Dolls from Nurse Dimsdale, Toby, Nurse Love, Miss Martin, Nurse Winterton.

Miss Warner, Panel; Mater, Velvet Bag, &c.; Lucy, Scrap Book; Miss Kindermann, Scrap Book; Ostrich, Scrap Book; A. M. E., Infant's Cape; Endive, Child's Socks and Jacket.

### Acrostic Competition.

A PRIZE OF ONE GUINEA will be given to the competitor who gains the highest number of marks for best original acrostics during the ensuing quarter. All acrostics sent in for this competition will be credited according to merit, twelve marks being the highest score given for one acrostic.

A PRIZE of HALF-A-GUINEA to be given to the competitor who gains the highest score for solution of acrostics set, including the one inserted on November 26th. One mark only will be given to the competitors who solve all the lights of each acrostic.

These competitions to alternate weekly.

This week credit will be given for No. 5 Double Original Acrostic in verse.

### Results of No. 4 Original Acrostic.

Pasteur (12), A. M. E. (12), Mater (12), Brownie (12), Ellice (12), G. H. (10), Gretta (9), Padre (9), Mar (7), Condorcet (6).

### II. Word and Literary Competition.

Commenced November 5th, 1887; ends January 28th, 1888.

Three prizes of one guinea, 15*s.* and 10*s.* 6*d.* will be given each quarter to the three competitors who obtain the highest number of marks: (1) by making the largest number of words derived from the word or phrase set for anatomical dissection; or (2) for the best answers to (6); or (3) by (1) and (2) combined.

(A) THE WORD COMPETITION.

We shall give the newspapers for dissection during this quarter, that for this, the last week of the quarter, being

"SPECTATOR."

OBSERVE.—Proper names, abbreviations, foreign words, words of less than four letters and repetitions are barred. Nuttall's *Standard* dictionary only to be used.

### Results of Eleventh Week.

*The Fortnightly.*—Ellice (389), Gretta (388), Reldas (373), E. E. (357), Brisbane (332), Lauriston (325), Tabs (294), Kath (280), Condorcet (269), K.M. (226).

(B) THE LITERARY COMPETITION.

Some people hate puzzles of all kinds, including word competitions, but they are fond of correspondence, and many sisters and mothers are continually receiving and sending letters to and from members of the family who are abroad in India or the Colonies. The constant coming and going between England and other portions of the British Empire make it a matter of interest and importance to know something about the life, the climate, the amusements, the adventures, the clothing, and the surroundings of those who seek a livelihood beyond the seas. We shall therefore try to find space for the publication of selected letters from abroad, which may be sent by any of our readers who have friends and relations in foreign lands. We shall give marks for these letters as well as for the word competitions which will count equally for these prizes, so that anyone may enter for both or either, the prizes to be given to those who get the highest number of marks.

Madre sends us a letter this week from Gibraltar, Gretta one from China.

### TRAVEL MADE EASY.

There are few people to whom the name of the P. and O. is not familiar. To many families in England it has long been a household word; indeed, few will say that Leadenhall-street has diminished in importance, even since the days when the East India Company rendered it famous. No greater proof of the vitality of this veteran company is needed than the vigour with which it appears to be entering upon the present year, from which its new mail contracts to India, China, and Australia date. These contracts commence in February, and the occasion has been availed of to re-model all the services. The public now-a-days demand greater facilities and greater comfort in travel than contented them formerly, and this demand has been met by vast improvements in all directions. The Government has decided that the mail services shall still be carried on by way of Brindisi, and the route through the Suez Canal being selected, there will no longer be any transhipments or railway transit across Egypt, which formerly was so annoying to travellers; neither will there be a recurrence of the vexatious delays and inconveniences to which they were at times subjected by the action of the sanitary authorities in regard to the imposition of quarantine in Egypt. Every Monday morning the letters from the East will be delivered as heretofore, and letters for the East will be dispatched, as at present, every Friday evening. But the conveyance of the mails to India, China, and Australia, and the British possessions in the East, is only a portion of the operations carried on by the P. and O. Company, and the directors have not been content to confine their services from London or Brindisi, but have also notified extensions from London to Marseilles and Naples, and from Marseilles, Naples, and Brindisi to all ports embraced in their Eastern system. They are also opening a service from Brindisi to Malta, which will bring this important and imperial naval and military station within 87 hours from London. But familiar as the name of the P. and O. is, few people are cognisant of the enormous growth of the fleet of this company, and that in it the Government possesses in reserve a naval power of great magnitude. At the present time the P. and O. Company owns 53 steamers, with an aggregate registered tonnage of 204,183 tons, and 198,500 horse-power, built at a cost of some £6,000,000. Their largest steamers are of 6,500 tons register, and the smallest 2,000 tons. Their vessels are not designed to maintain a high rate of speed at sea for short voyages only, but for lengthened periods, and they are all of the highest class, being specially built for the conveyance of mails and passengers (cargo taking of necessity a more or less subsidiary position). They are all admirably adapted to the carriage of troops, of which it is interesting to know they could convey, according to the Admiralty rules of measurement, no less a number than 54 000 men. Their largest ship can easily convey 2,650 men, and their smallest 1,000 men, besides officers; and it will be remembered that during the Indian Mutiny and Crimean War, and in the recent Egyptian Campaign, the Company's ships carried a large number of troops for the Government without a single casualty. The magnitude of this great national company's operations becomes more apparent when we consider that in the course of the coming year no less than 200 of their steamers, of an aggregate tonnage of nearly 1,000,000 tons, will enter and leave the port of London alone. The steamers traverse 2,500,000 miles in the course of a year, and the Company gives employment to 800 officers (commanders, officers, engineers surgeons, etc.) of the mercantile marine, holding certificates from the Board of Trade, and a large proportion have her Majesty's commission in the Royal Naval Reserve. It has also in its service an army of nearly fifteen thousand people afloat and ashore, and no less a sum than £2,000,000 sterling is expended every year in coals, wages, stores, and provisions. It also possesses a dockyard in Bombay worthy of a great naval power, and coaling stations at all the principal ports of the East, which in time of emergency have often proved useful to the Government in the past, and are likely to do so in the future. Gigantic though its resources and operations are, the old Company has many competitors, and although its main operations are directed towards the carriage of mails and passengers, the competition of the cheap class of cargo steamers, with limited passenger accommodation, which has lately been built, and with which the trades of the world are so fully supplied, has forced the Company to extend its branches, and of late it has established a network of agencies throughout the country. It is a matter for congratulation that the country has at the head of one of its greatest national enterprises an enormous organization such as this, and that while its existence must ever guarantee a reserve of power to our Navy and Army, it is ceaselessly and actively engaged night and day in furthering the interests of peace, and knitting together the peoples of the East and West.

### THE CHILDREN'S CORNER.

PRIZES.

FOR MOST CORRECT ANSWERS TO PUZZLES.

This Commenced October 1st, 1887.

One Pound a Month.

A PRIZE OF THREE GUINEAS

to the Competitor who shall have sent in the largest number of correct or best answers during the twelve months.

### Puzzles—Fourth Week.

No. 13. RIDDLE-ME-REE.

My *first* is in cow, but not in horn,
My *second* is in pro, but not in con,
My *third* is in swallow, but not in swift,
My *fourth* is in waste, but not in thrift,
My *fifth* is in rent, but not in rift.

No. 14 ARITHMETICAL PUZZLE.—Three women went to market with oranges. The *first* had 50, the *second* 30, the *third* 10. All three sold their oranges at two different prices, which were the same in each case, and after selling all their fruit they each obtained the same amount of money. How was this?

No. 15. ENIGMA.

I have an arm without a bone,
A back without a spine,
No legs, a tail together place,
And say what name is mine.

No. 16. GEOGRAPHICAL CHARADE.—1. A town in France. 2. An island in the Mediterranean Sea. 3. A lake in Scotland. 4. A town in Surrey. 5. A river in South America. 6. A seaport town in Sussex. My *primals* will give you the name of an English river, and my *finals* what is found in it.

### Results of Second Week Puzzles.

No. 5. GEOGRAPHICAL CONUNDRUM.—The Scotch river often mentioned in Spain is the *Don*.

No. 6. SQUARE WORD.

H A N D
A G U E
N U T S
D E S K

No. 7. HISTORICAL ACROSTIC.

H ohenlinden.
A gincourt.
S t. Albans.
T ewkesbury.
I reland.
N eville's Cross.
G lencoe.
S purs.

No. 8. ENIGMA.—A Hat.

*All right*—Happy Eliza, Toad, Judy, A.C.K., Druce, Jumps, Plummy, Fluff, P.S., Scrooge, Robinson, Excelsior. *Three right*—Pepita, Spider, Jacko, Gem, Tim. *Two right*—A. G. S. McCulloch. *One right*—Fern, Poodle.

**Notice to all Correspondents.**—N.B.—All letters referring to this page, which do not arrive at 38, Old Jewry, Cheapside, London, E.C., by the *first post on Thursdays*, and are not addressed PRIZE EDITOR, will in future be disqualified and disregarded.

No one will be permitted to win the Monthly or Quarterly Prizes twice in any one year, the annual prizes being offered especially to prevent this.

